# Reliability of thoracolumbar burst fracture classification in the Swedish Fracture Register

**DOI:** 10.1186/s12891-024-07395-0

**Published:** 2024-04-12

**Authors:** Simon Blixt, Fabian Burmeister, Sebastian Mukka, Lukas Bobinski, Peter Försth, Olof Westin, Paul Gerdhem

**Affiliations:** 1 Department of Orthopaedics and Hand surgery, Uppsala University Hospital, Uppsala, Sweden; 2 Department of Surgical Sciences, Uppsala University, Uppsala, Sweden; 3 Department of Clinical Science Intervention and Technology, Karolinska Institutet, Stockholm, Sweden; 4 Department of Surgical and Perioperative Sciences (Orthopaedics), Umeå University, Umeå, Sweden; 5 Department of Orthopaedics, Institute of Clinical Sciences at Sahlgrenska Academy, University of Gothenburg, Gothenburg, Sweden; 6 Spine Surgery Unit, Orthopedic Clinic, Sahlgrenska University Hospital, Gothenburg, Sweden

**Keywords:** Accuracy, Agreement, Burst fracture, Classification, Register-based, Reliability, Thoracolumbar

## Abstract

**Background:**

The Swedish Fracture Register (SFR) is a national quality register for all types of fractures in Sweden. Spine fractures have been included since 2015 and are classified using a modified AOSpine classification. The aim of this study was to determine the accuracy of the classification of thoracolumbar burst fractures in the SFR.

**Methods:**

Assessments of medical images were conducted in 277 consecutive patients with a thoracolumbar burst fracture (T10-L3) identified in the SFR. Two independent reviewers classified the fractures according to the AOSpine classification, with a third reviewer resolving disagreement. The combined results of the reviewers were considered the gold standard. The intra- and inter-rater reliability of the reviewers was determined with Cohen’s kappa and percent agreement. The SFR classification was compared with the gold standard using positive predictive values (PPV), Cohen’s kappa and percent agreement.

**Results:**

The reliability between reviewers was  high (Cohen’s kappa 0.70–0.97). The PPV for correctly classifying burst fractures in the SFR was high irrespective of physician experience (76–89%), treatment (82% non-operative, 95% operative) and hospital type (83% county, 95% university). The inter-rater reliability of B-type injuries and the overall SFR classification compared with the gold standard was low (Cohen’s kappa 0.16 and 0.17 respectively).

**Conclusions:**

The SFR demonstrates a high PPV for accurately classifying burst fractures, regardless of physician experience, treatment and hospital type. However, the reliability of B-type injuries and overall classification in the SFR was found to be low. Future studies on burst fractures using SFR data where classification is important should include a review of medical images to verify the diagnosis.

**Supplementary Information:**

The online version contains supplementary material available at 10.1186/s12891-024-07395-0.

## Background

Burst fractures are compression fractures of the spine involving injury to the posterior wall of the vertebral body and at least one end plate [1]. They often occur in the thoracolumbar transition due to the biomechanical forces being highest in this area [2].

The Swedish Fracture Register (SFR) is a nationwide quality register that collect data on all types of orthopedic fractures [3]. Data collected include the date and cause of injury, fracture classifications, treatment, reoperations, and patient-reported outcome measures. The SFR uses a simplified AO/OTA classifications [4] with the aid of pictures to guide the registering physician. Registrations in SFR are made by physicians of different levels of experience, including interns, residents, emergency physicians and orthopedic surgeons.

Spine fractures have been included in the SFR since 2015 [5]. As physicians of any expertise may register the fracture, the accuracy of the classifications, and in turn, the reliability of the data in the SFR may be compromised.

## Methods

### Aim

The aim of this study was to determine the reliability of the classification of thoracolumbar burst fractures in the SFR.

### Study design

This study is retrospective on prospectively collected data.

### Study population

Patients in working age, 18–66 years, with a single-level thoracolumbar fracture from the tenth thoracic vertebra to the third lumbar vertebra classified as a burst fracture were identified in the SFR.

Data in the SFR during the time period included the majority of trauma and orthopedic departments in Sweden, including university hospitals and county hospitals [3]. Data is entered by the physician treating the patient in the web based platform, often by specialist or resident in orthopedics [5], but may be registered by even less experienced physicians [6]. Registrations is optimally entered already in the emergency department, but subsequent treatments can be added later [6]. Details concerning the registration process are described in the Appendix.

Thoracic and lumbar fractures in the SFR are classified using a modified version of the 2013 version of AO spine injury classification (AOSpine classification) by Reinhold et al. [7]. The physician selects the fracture level, neurological function, and the type of fracture with the assistance of pictures and information by text. As opposed to the original classification, the SFR classification doesn’t distinguish between incomplete and complete burst fractures [8]. Compression fractures are divided in three categories: simple compression fractures (A1), pincer fractures (A2) and burst fractures (A3/4). The physician then asked to add whether there is a concomitant injury to the posterior tension band (B-type injury). Finally, the physician is asked to decide if there are signs of ankylotic spinal disorder in the fractured area. A detailed description of the modified AOSpine classification is available in the Appendix.

**Fig. 1:**
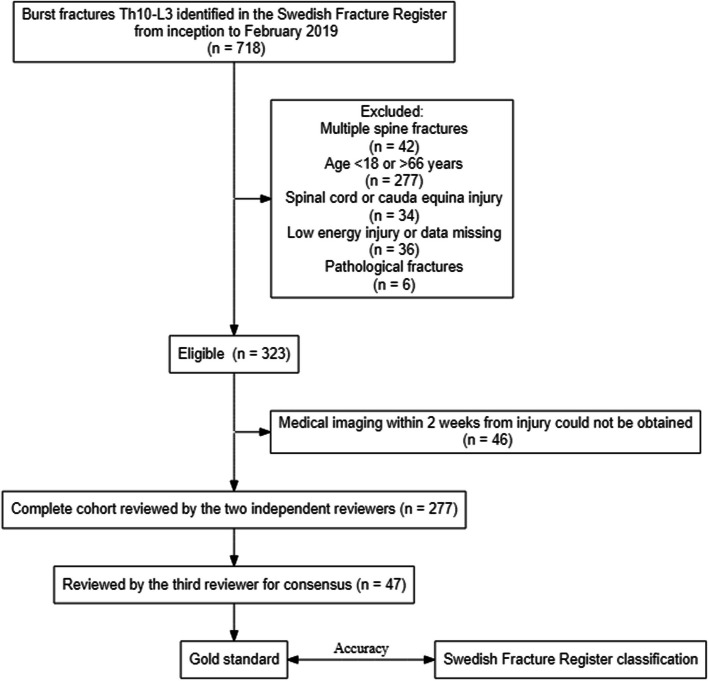
Flow chart of the study

The flow chart of the study is presented in Fig. 1. In this study, patients with factors that influence the decision for or against operative treatment were excluded. These factors included injury to the spinal cord or cauda equina, open fractures, pathological fractures, fractures on multiple levels and low-energy injuries (defined as a fall from standing height or less). Patients were collected from the inception of spine fracture registrations in the SFR, starting in 2015, until February 2019.

Patients with any type of medical imaging within two weeks from the time of injury, as well as before surgery for the operatively treated patients, were eligible for the study. Computed tomography (CT), magnetic resonance imaging (MRI) or conventional radiograph of the spine were collected from the treating hospitals. If more than one modality was available, CT and MRI were prioritized.

### Medical imaging assessment

Medical images were reviewed and classified independently by two physicians (SB and FB) and by a third physician (PG) where disagreement on classification occurred. The radiographs were anonymized, and the reviewers only had information about the patient’s birthdate and treatment. No information about patient history, cause of injury, sex and the radiologist evaluation were available. The fracture level was identified, and the fracture type was classified according to the AOSpine classification by Reinhold et al. [7]. The three reviewers had different levels of experience (SB orthopedic resident with training in the AOSpine classification, FB specialist in orthopedic surgery and PG specialist in orthopedics and experienced spine surgeon). The combined results of the three reviewers were considered the gold standard.

### Agreement

To evaluate the intra-rater reliability, the reviewers SB and FB assessed a subset of 52 patients, 3 to 6 months after the first classification. For assessing the inter-rater reliability between reviewers SB and FB the initial classification was used. Finally, the inter-rater reliability between the gold standard and the SFR classification was performed.

### Statistics

Data are presented with means, medians, quantiles, minimum, maximum and standard deviations for continuous variables, and numbers and percentages for categorical variables. The intra- and inter-rater reliability was determined with percentages of agreement and weighted Cohen’s kappa. The reliability was interpreted using the criteria first proposed by Landis and Koch [9], with kappa coefficients of 0.0–0.2 representing slight reliability, 0.2–0.4 as fair, 0.4–0.6 as moderate, 0.6–0.8 as substantial, and > 0.8 as excellent. The positive predictive value (PPV) was calculated to determine the accuracy of the classifications. Comparisons were made based on experience of the registering physician, choice of treatment (operative vs. non-operative), and hospital type. Statistical significance was set to *p* < 0.05.

RStudio software version 4.1.0 for Windows (R Foundation for Statistical Computing) was used for all statistical analyses.

## Results

### Patients and descriptive data

The patient characteristics are summarized in Table 1.

**Table 1:** Patient characteristics

**Variable**	
*n*	277
*Age (years)*	
Mean (SD)	43.1 (14.6)
*Sex*	
Female	125 (45%)
Male	152 (55%)
*Medical imaging modality reviewed, n (%)*	
Conventional radiograph	11 (4%)
CT	206 (74%)
CT and MRI	56 (20%)
MRI	4 (1%)
*Registering physician in the SFR, n (%)*	
Intern	16 (6%)
Orthopedic surgeon	43 (16%)
Resident	36 (13%)
Spine surgeon	123 (44%)
Unknown/Missing	59 (21%)
*Mechanism of injury, n (%)*	
Bicycle accident	6 (2%)
Exposure to mechanical forces	7 (3%)
Fall from height	126 (45%)
Horse riding accident	26 (9%)
Road traffic accident	91 (33%)
Self-inflicted injury	10 (4%)
Struck against object	6 (2%)
Watercraft related accident	1 (0%)
Missing	4 (1%)
*Treatment, n (%)*	
Non-operative	167 (60%)
Operative	110 (40%)
*Fracture level SFR, n (%)*	
Th10	3 (1%)
Th11	11 (4%)
Th12	64 (23%)
L1	126 (45%)
L2	45 (16%)
L3	28 (10%)
*SFR type A, n (%)*	
A3/4^a^	277 (100%)
*SFR type B, n (%)*	
B0	148 (53%)
B1	52 (19%)
B2	38 (14%)
BX	39 (14%)
*Neurology, n (%)*	
Intact	269 (97%)
Radiculopathy	8 (3%)

*SFR* Swedish Fracture Register, *Fracture level SFR* Fracture level as registered in the SFR, *SFR class A* A-type fracture as registered in the SFR, *SFR class B* B-type fracture as registered in the SFR, *B0* No injury to the posterior tension band, *B1* Fracture through vertebral body and rupture of the posterior tension band through bone, *B2* Rupture of the posterior tension band with or without skeletal injury, *BX* Injury to the posterior tension band can’t be determined

^a^The modified AOSpine classification in the SFR does not separate incomplete and complete burst fractures

Comparisons between the SFR classification and the gold standard classification are displayed in Table 2. The accuracy and agreement of the fracture level in comparison to the gold standard was excellent (95%). The number of A1 compression fractures misclassified as a burst fracture was significantly greater for non-operatively treated patients compared to operatively treated (25 (15%), 3 (3%) respectively). There was a significant difference in experience in registrations between operatively and non-operatively treated patients with the majority of operatively treated patients being registered by a spine surgeon (85%), while the non-operative patients were registered by physicians of varying level of experience.

**Table 2:** Comparison between gold standard and SFR

	**Gold standard**	**SFR**	**Gold standard**	**SFR**	**Gold standard**	**SFR**
	Overall		Non-operative		Operative	
	277		167		110	
Th10	3 (1%)	3 (1%)	2 (1%)	2 (1%)	1 (1%)	1 (1%)
Th11	8 (3%)	11 (4%)	5 (3%)	7 (4%)	3 (3%)	4 (4%)
Th12	63 (23%)	64 (23%)	45 (27%)	44 (26%)	18 (17%)	20 (18%)
L1	125 (45%)	126 (45%)	68 (41%)	68 (41%)	57 (53%)	58 (53%)
L2	48 (17%)	45 (16%)	27 (16%)	26 (16%)	21 (19%)	19 (17%)
L3	28 (10%)	28 (10%)	20 (12%)	20 (12%)	8 (7%)	8 (7%)
Burst fracture	241 (87%)	277 (100%)	137 (82%)	167 (100%)	104 (95%)	110 (100%)
Not burst fracture	36 (13%)	0 (0%)	30 (18%)	0 (0%)	6 (5%)	0 (0%)
A1	28 (10%)	0 (0%)	25 (15%)	0 (0%)	3 (3%)	0 (0%)
A2	4 (1%)	0 (0%)	4 (2%)	0 (0%)	0 (0%)	0 (0%)
A3/4	241 (87%)	277 (100%)	137 (82%)	167 (100%)	104 (95%)	110 (100%)
A3	115 (42%)	-	87 (52%)	-	28 (25%)	-
A4	126 (45%)	-	50 (30%)	-	76 (69%)	-
Other	4 (1%)	0 (0%)	1 (1%)	0 (0%)	3 (3%)	0 (0%)
B0	233 (84%)	148 (53%)	159 (95%)	120 (72%)	74 (67%)	28 (25%)
B1	1 (0%)	52 (19%)	0 (0%)	10 (6%)	1 (1%)	42 (38%)
B2	43 (16%)	38 (14%)	8 (5%)	9 (5%)	35 (32%)	29 (26%)
BX	0 (0%)	39 (14%)	0 (0%)	28 (17%)	0 (0%)	11 (10%)
C1	2 (1%)	0 (0%)	1 (1%)	0 (0%)	1 (1%)	0 (0%)
C2/3	1 (0%)	0 (0%)	0 (0%)	0 (0%)	1 (1%)	0 (0%)

*SFR* Swedish Fracture Register, *Gold standard* Combined classification by the reviewers, *A1* Wedge compression, *A2* Pincer type compression, *A3/4* Burst fracture without specification, *A3* Incomplete burst fracture, *A4* Complete burst fracture, *Other* Unable to classify, *B0* No injury to the posterior tension band, *B1* Fracture through vertebral body and rupture of the posterior tension band through bone, *B2* Rupture of the posterior tension band with or without skeletal injury, *BX* Injury to the posterior tension band can’t be determined, *C1* Hyperextension injury without translation (injury to the anterior part of the vertebral column through the disc or vertebral body in a hyperextension position) *C2/3* Translation injury or dislocation injury through bone or disc/ligament

### Intra- and inter-rater reliability

The intra- and inter-rater reliability of the reviewers are presented in Table 3. The percentage of agreement comparing classifications made by the reviewers on different occasions were excellent (83 − 99%). The intra-rater reliability was substantial to excellent, with the exception for B type injuries for reviewer 2 which had fair reliability (kappa 0.28).

**Table 3:** Intra and inter-rater reliability of the reviewers presented as percentage of agreement (PA) and Cohen´s kappa

	**Intra-rater reliability**				**Inter-rater reliability**			
	**Reviewer 1**		**Reviewer 2**		**Reviewer 1**		**Reviewer 2**	
	PA	Kappa	PA	Kappa	PA	Kappa	PA	Kappa
Type A	94%	0.85 (0.09)	92%	0.93 (0.03)	96%	0.91 (0.03)	87%	0.78 (0.04)
Type A (SFR classification)	94%	0.78 (0.12)	98%	0.97 (0.03)	97%	0.86 (0.05)	92%	0.70 (0.06)
Type B	96%	0.80 (0.14)	90%	0.28 (0.16)	97%	0.92 (0.03)	99%	0.97 (0.02)
AOSpine classification	92%	0.83 (0.09)	83%	0.61 (0.12)	93%	0.93 (0.02)	86%	0.91 (0.02)
SFR classification	90%	0.81 (0.11)	88%	0.52 (0.12)	95%	0.90 (0.03)	91%	0.88 (0.03)

*Intra-rater reliability* Comparing reviewers with themselves, *Inter-rater reliability* Comparing reviewers with gold standard, *ASD* Asymptotic standard error, *PA* Percentage of agreement, *Type A* AOSpine type A injury (A1, A2, A3, A4 or other), *Type A (SFR classification)* AOSpine type A injury using the SFR simplification (A1, A2, A3/4 or other), *Type B* AOSpine type B injury (B0, B1, B2), *AOSpine classification* The combined AOSpine classification of A and B injuries, *SFR classification* The combined AOSpine classification using the simplification where A type fractures are divided in simple compression (A1), pincer (A2) and burst (A3/4)

### Third review assessment

Of the total of 277 patients, 71 patients were assessed by the third reviewer. 10 patients were reviewed due to disagreement on the fracture level. 47 patients were reviewed for disagreement in the classification of type A-injury. Among these, 19 cases were reviewed for disagreement whether there was a wedge compression (A1) or an incomplete burst fracture (A3), and 17 cases for whether there was an incomplete (A3) or a complete (A4) burst fracture. The remaining 11 cases had disagreement whether there was a simple compression (A1) or a pincer fracture (A2) (3 patients), wedge compression (A1) or a complete burst fracture (A4) (3 patients), a pincer fracture (A2) or a complete burst fracture (A4) (1 patient), a fracture that should not be classified as A-type (4 patients). 8 (of 277) patients were reviewed for disagreement in the classification of type B-injures.

The inter-rater reliability between reviewers and gold standard were substantial to excellent across all sub-classifications (Table 3).

### Comparison between SFR and the gold standard

#### Accuracy

The accuracy determined by the PPV for correctly classifying patients having a burst fracture remained high regardless of the experience of the physician, for non-operatively and operatively treated patients and type of hospital (Table 4). However, the classification of B-type injuries was notably less accurate with PPV ranging from 0 to 40%.

**Table 4:** Accuracy presented as PPVs comparing SFR with gold standard

**Group**	**n**	**PPV type A3/4 (burst fracture)**	**PPV type B1**	**PPV type B2**	**PPV type B without subtypes**
All	277	87%	2%	21%	36%
Spine surgeon	123	89%	3%	27%	39%
Non-spine surgeon	154	85%	0%	0%	25%
- Orthopedic surgeon	43	84%	0%	0%	33%
- Resident	36	86%	0%	0%	0%
- Intern	16	75%	-	0%	0%
- Unknown/Missing	59	88%	0%	0%	33%
Operative	110	95%	2%	21%	38%
Non-operative	167	82%	0%	22%	26%
University Hospital	128	91%	2%	24%	40%
County Hospital	149	83%	0%	15%	22%

*PPV* positive predictive value, *A3/4* Burst fracture, *B1* Fracture through vertebral body and rupture of the posterior tension band through bone, *B2* Rupture of the posterior tension band with or without skeletal injury, *B without subtypes* Ignoring the subtypes of B injuries. All B1 and B2 injuries are classified as B

#### Agreement between the SFR with the gold standard

The percentage of agreement and inter-rater reliability of the SFR classifications compared with the gold standard are summarized in Table 5. The estimation of Cohen’s kappa for A-type fractures was not possible for this dataset, as all reviewed fractures were determined to be burst fractures according to the SFR. The reliability of the fracture level was excellent (kappa 0.94). The reliability for B-type injuries were only slight (Cohen’s kappa 0.15). When ignoring the subtype of B injury, the reliability slightly improved (kappa 0.34). The overall reliability of the AOSpine classification with the SFR modification was slight (Cohen’s kappa 0.08) even when ignoring the subtype of B-injury registered (kappa 0.23). The inter-rater reliability did not change when analyzing subgroups by age, sex, treatment, registering physician or fracture severity (A3 vs. A4).

**Table 5:** Inter-rater reliability presented as percentage of agreement (PA) and Cohen´s kappa between the gold standard and SFR-classification

AO classification	PA	Kappa (ASD)
Fracture level	96%	0.96 (0.01)
Type A (SFR classification)	87%	-
Type B	69%	0.16 (0.04)
Type B without subtypes	75%	0.34 (0.06)
AOSpine classification (SFR-modification)	45%	0.17 (0.03)
AOSpine classification (SFR-modification) without B-subtypes	53%	0.31 (0.05)

*ASD* Asymptotic standard error, *PA* Percentage of agreement, *Type A (SFR classification) *AO type A injury using the SFR simplification (A1, A2, A3/4 or other), *Type B *AO type B injury (B0, B1, B2, BX), *Type B without subtypes *AO type B injury (B0, B), *AOSpine classification (SFR-modification) without B-subtypes* Without subtypes of B-injuries

## Discussion

The accuracy of correctly classifying a burst fracture in the SFR is excellent regarding level of fracture and type A injury regardless of the experience of the registering physician. However, the classification of B-type injuries in the SFR is far less reliable, with a significant number misclassification even among spine surgeons.

The SFR, being a national quality register that collects data on all types of orthopedic fractures, is a valuable resource for observational research on patients with thoracolumbar burst fractures to identify factors associated with treatment outcomes. Furthermore, as SFR expands to register-based randomized controlled trials, it is important that the classification of burst fractures is reliable [10]. The present study focused on investigating the reliability of classifications for thoracolumbar burst fractures in selected patients where both operative and non-operative options both are considered valid. This is a group of patients that may be registered and classified by physicians with a wide range of experience based on the local tradition of how they are usually treated.

The intra-rater and inter-rater reliability of the AOSpine classification as assessed by the reviewers in our study demonstrated substantial to excellent reliability. This is in accordance with previous reliability studies made on the classification system which have shown moderate to substantial reliability [11–14]. In a previous study by Morgonsköld et al., the reliability of spine fractures in the SFR was considered acceptable with moderate agreement [8]. In the study, physicians of varying experience levels were involved, all of whom had previous knowledge of the different classification systems used in the SFR, including AOSpine classification. Their gold standard was determined through the consensus of two experienced physicians and accuracy was measured using Cohen’s kappa. However, no direct comparison was made with the recorded classifications in the SFR. In practice, fractures may be registered by physicians without specific training in the classification systems being used.

Based on our results the likelihood that a burst fracture is correctly registered as a burst fracture in the SFR is high regardless of the level of experience in the registering physician. Although the percentage of agreement and PPV was high amongst all levels of experience, the number of simple compression fractures was substantially larger for non-operatively treated patients. Most registrations of operatively treated spine fractures were classified by surgeons themselves, whereas non-operatively treated spine fractures were registered by physicians of varying expertise, which explains the difference. In addition, the proportion of complete burst fractures (A4) compared to incomplete (A3) was higher in the operative group, which is also reasonable, as surgeons are likely to be more inclined to choose operative treatment options in patients with more severe fractures. Because the SFR classification do not distinguish between incomplete and complete burst fractures, simple comparisons between treatment groups may be erroneous, as the groups are not completely similar. The correct identification of complete burst fractures (A4) has been demonstrated to be challenging even among spine surgeons [14]. Consequently, opting not to separate burst fractures into incomplete and complete in the SFR is reasonable, considering the wide range of expertise among the registering physicians.

However, it is necessary to draw attention to the inter-rater reliability for B-type injuries, which was comparatively lower when comparing the gold standard with the classification in the SFR. Previous studies have shown that the inter-rater reliability of posterior tension band injuries tends to be lower compared to type A and C injuries [15–17]. Surprisingly, our study demonstrated an even lower level of reliability in this respect. A significant number of the fractures were misclassified as B1. This discrepancy may be explained by the accompanying textual descriptions with the pictures in the SFR that describes B1 as a “fracture through the vertebral body and rupture of the posterior tension band structures through bone” and B2 as “Rupture of the posterior tension band with or without skeletal injury”. It may not immediately apparent that a B1 fracture signifies a monosegmental osseous failure to a physician without knowledge of the classification system. This finding was also unexpectedly common for spine surgeons who should be familiar with the concept of the posterior tension band. According to our results, most physicians have registered any skeletal injury to the posterior structures of the lamina, including non-distraction type injuries such as vertical laminar fractures, as B-injuries. It should be noted that B3 type injuries according to the latest version of the AOSpine classification [16] is classified as C1 in the SFR classification, similarly to the classification presented by Reinhold et al. [7]. The absence of B3 injuries in our material can be explained by the fact that we excluded patients with ankylosing disorders given their increased likelihood of presenting with unstable fractures necessitating surgical fixation [18], and considering that the distinct injury mechanism associated with B3 injuries are primarily characterized by hyperextension [16] in contrast to axial compression for burst fractures [1].

To improve the reliability of B-type injury classifications in the SFR, we suggest that the register consider adding further choices with images depicting various types of fractures to the lamina, including vertical and horizontal spinous fractures. Additionally, it could prompt users to specify whether the horizontal spinous fracture is at the same level as the vertebral body fracture or not. These enhancements may help reduce misclassifications, ultimately improving the reliability of data collected in the SFR.

For thoracolumbar fracture to be reliably classified a CT is the imaging modality of choice [19]. In Swedish medical care where a fracture of the spine is considered a CT is the modality performed and conventional radiography has more or less become obsolete. The SFR doesn’t specify which imaging modality has been used for making the diagnosis. In our material most patients had undergone a CT, which was expected. 11 patients only had a conventional radiography. We chose to not exclude the patients with only conventional radiography as we aimed at determining the reliability of thoracolumbar burst fractures in the register. Excluding the cases with only a conventional radiography may have improved the reliability slightly. In 4 cases we only retrieved an MRI at the time of injury. In all 4 cases the patients underwent surgery and postoperative CT where available. We expect these cases to be patients from smaller hospitals that had been referred to the university hospital, although we have no possibility to verify this assumption. We chose to include these patients as the MRI was sufficient to determine whether a burst fracture was present or not, even though MRI is not the modality of choice determining skeletal injury [19].

In our study, most patients had not undergone an MRI within two weeks of injury. MRI plays an important role regarding soft tissue injuries and previous studies have shown that the classification of thoracolumbar fractures can change in 10 to 30% of cases [19–23]. The limited utilization of MRI may have implications for the accuracy of the true fracture classification, especially regarding B-type injuries although the clinical implications regarding a change in treatment with routine MRI are still questionable [19, 23].

### Strengths

The collection of consecutive patients from multiple centers and comparison with the classifications made in the SFR by physicians of different levels of experience under real-life conditions constitute the strengths of this study.

### Limitations

There are some limitations of this study that should be acknowledged. The cohort was prospectively collected but the analysis and classification of medical images was carried out retrospectively. We did not have clinical information about the patients, such as symptoms, levels of pain and comorbidities. Only burst fractures were collected and reviewed, which meant that the inter-rater reliability of A type injuries between the gold standard and SFR could not be made. We only reviewed selected patients where both operative and non-operative treatment are both valid options.

The patients identified for this study are likely to be only a fraction of the true number of patients in Sweden with a thoracolumbar burst fracture during this period. Since then, the coverage of SFR has increased SFR [6]. Despite this limitation, our study provides valuable insights to the SFR’s classification reliability of thoracolumbar burst fractures, emphasizing the need for further considerations in future research in this area.

## Conclusions

The accuracy of classifying thoracolumbar burst fractures in the SFR is high regardless of the level of experience by the physician, treatment allocation and treating hospital. However, the inter-rater reliability of the AOSpine classifications of thoracolumbar burst fractures in the SFR is low when compared to reviewers with specific training in the classification system, particularly concerning B-type injuries. There are noticeable differences between operatively and non-operatively treated patients, and simple comparisons between treatments with data from the SFR without further review of medical images may lead to erroneous results. Future register-based studies on burst fractures with data from the SFR where classifications are of importance and when comparisons between operatively and non-operatively treated patients are made should include a review of medical images to verify the registered diagnosis.

### Supplementary Information

**Supplementary Material 1.**

**Supplementary Material 2.**

## Data Availability

The data used for the analyses in this manuscript are available in the supplementary material.

## Abbreviations

CT: Computed tomography
MRI: Magnetic resonance imaging
PPV: Positive predictive value
SFR: Swedish Fracture Register
