# Supplementary material for: Reliability of thoracolumbar burst fracture classification in the Swedish Fracture Register
Source: BMC Musculoskelet Disord. 2024 Apr 12;25:281. doi: 10.1186/s12891-024-07395-0 (PMC11010401; doi:10.1186/s12891-024-07395-0)
Supplement: Supplementary file 1 — Supplementary Material 1. [file 12891_2024_7395_MOESM1_ESM.docx]

**Appendix**

In the Swedish Fracture Register (SFR), vertebral fractures are divided into regions: cervical spine, thoracic spine, and lumbar spine. When registering a vertebral fracture, the damaged segment in the vertebral column is selected on a skeletal figure.


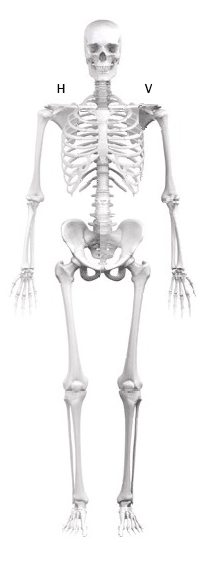

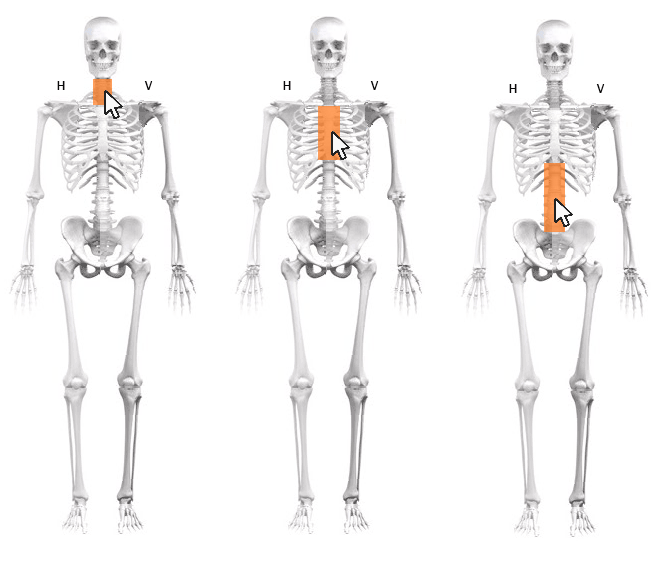


If multiple vertebrae are injured, the most severely affected vertebra or vertebral segment is classified. If a fracture occurs in more than one region (cervical spine, thoracic spine, or lumbar spine), registration of an additional fracture is required to classify this injury as well.

For cervical spine injuries, there are subgroups for fractures in the occipital condyle (C0), atlas (C1), axis (C2), or C3-Th1, based on commonly used classification systems (1-4). The classification of thoracic and lumbar spine fractures are based on the AO spine injury classification by Reinhold et al from 2013 (5), which is in turn based on the Magerl AO classification (6) with fractures grouped in A (compression), B (distraction), and C (displacement) type injuries. In addition to the fracture, the patient's neurology is also assessed.

For thoracic and lumbar fractures, the fracture level is selected first. You then have the option to select if the fracture should be regarded as an osteoporotic vertebral compression without certain trauma.


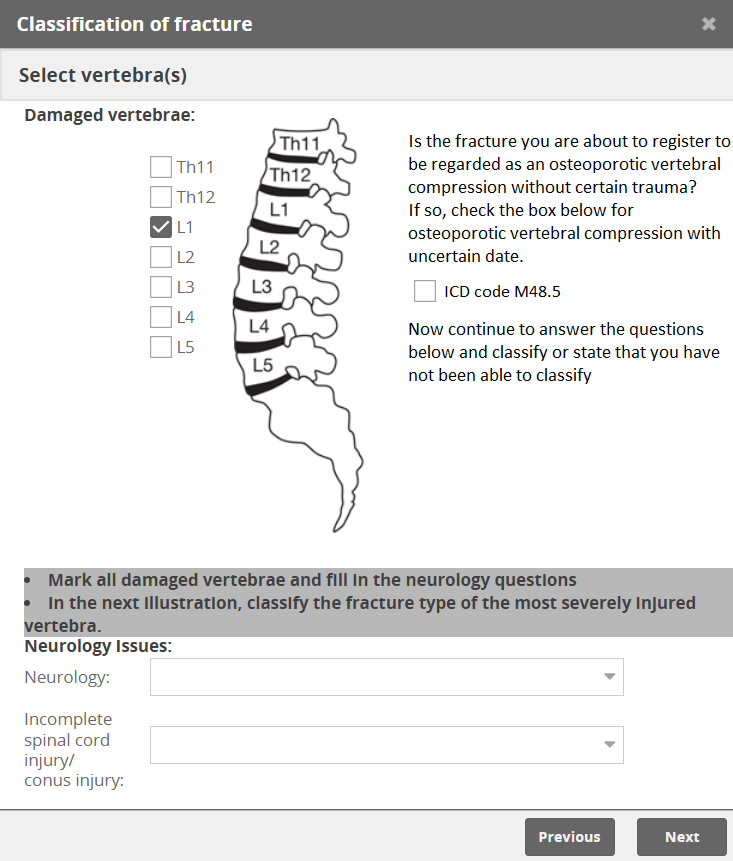


After the fracture level you register the neurological deficit as intact, nerve root damage, complete spinal cord/conus injury, incomplete spinal cord/conus injury, cauda equina impact, or not possible to assess.


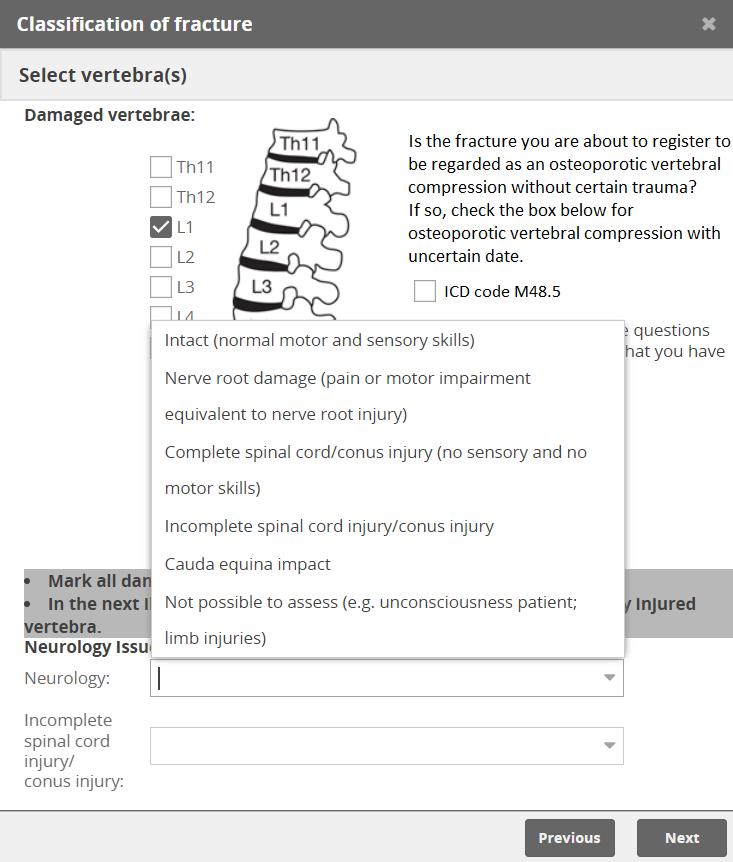


If the option incomplete spinal cord/conus injury is registered you can further describe the degree of injury according to the Frankel scale (7).


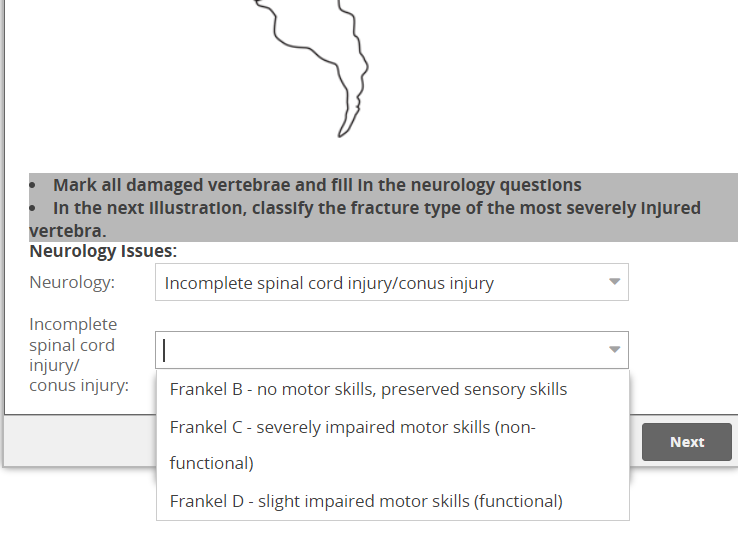


In the next step the fracture type is registered. You start by selecting the type of compression fracture (type A) or type of displacement fracture (type C). Distraction type injuries (type B) are selected in the next step.

The compression type injuries are subdivided into:

A1 – wedge-shaped compression

A2 – pincer-type fracture

A3/4 – burst fracture (both incomplete and complete)

The displacement type injuries are subdivided into:

C1 - Hyperextension injury without translation (injury to the anterior part of the vertebral column through the disc or vertebral body in a hyperextension position)

C2/3 - Translation injury or dislocation injury through bone or disc/ligament


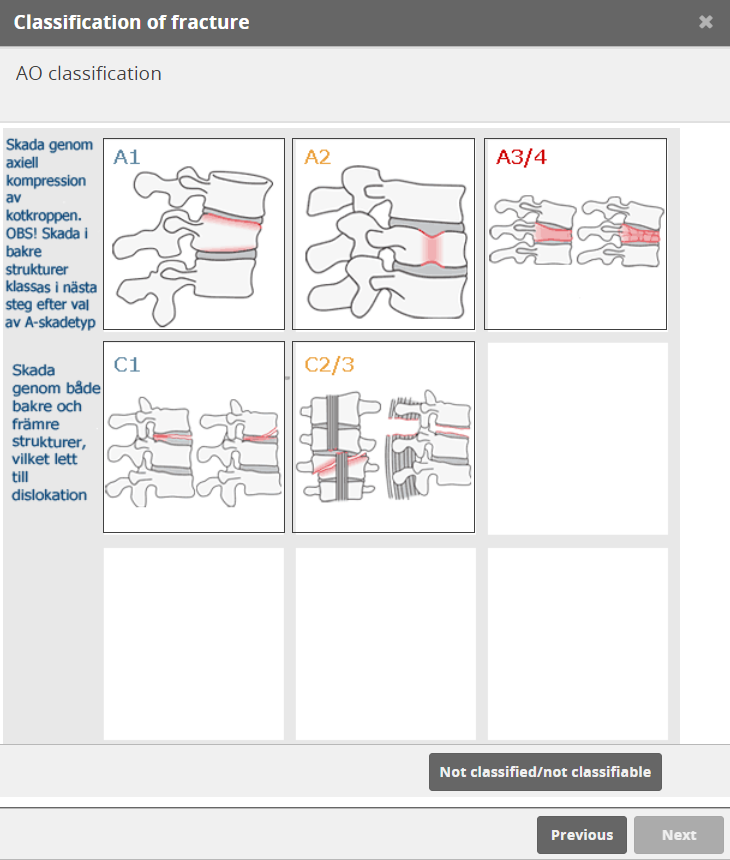


If a compression type injury is selected you are then able to register any type of distraction type injury (type B). The distraction type injuries are subdivided into:

B0 - No injury to posterior structures

B1 - Fracture through vertebral body and rupture of posterior tension band structures through bone (posterior)

B2 - Rupture of the posterior ligament with or without posterior skeletal injury

BX - Posterior injury cannot be assessed


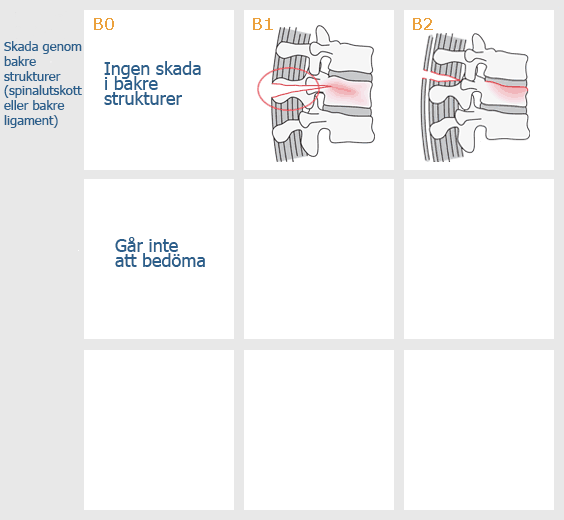


In the last step you select whether there are signs of diffuse idiopathic skeletal hyperostosis (DISH) or ankylosing spondylitis (Mb Bechterew) in the fracture area


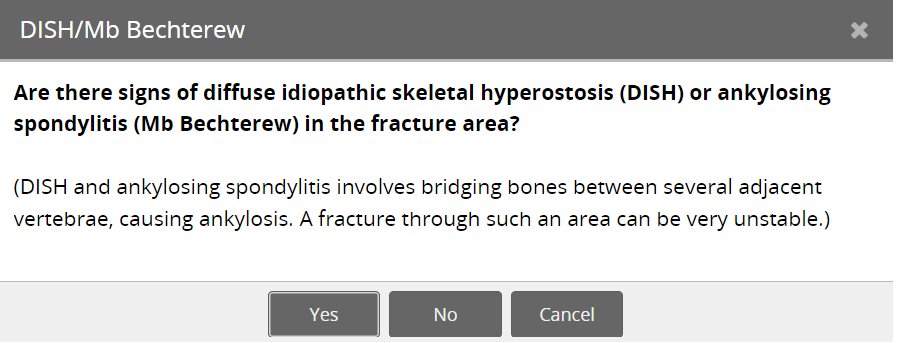


**References**

1. Anderson PA, Montesano PX. Morphology and treatment of occipital condyle fractures. Spine (Phila Pa 1976). 1988;13(7):731-6.

2. Jackson RS, Banit DM, Rhyne AL, 3rd, Darden BV, 2nd. Upper cervical spine injuries. J Am Acad Orthop Surg. 2002;10(4):271-80.

3. Anderson LD, D'Alonzo RT. Fractures of the odontoid process of the axis. J Bone Joint Surg Am. 1974;56(8):1663-74.

4. Vaccaro AR, Hulbert RJ, Patel AA, Fisher C, Dvorak M, Lehman RA, Jr., et al. The subaxial cervical spine injury classification system: a novel approach to recognize the importance of morphology, neurology, and integrity of the disco-ligamentous complex. Spine (Phila Pa 1976). 2007;32(21):2365-74.

5. Reinhold M, Audigé L, Schnake KJ, Bellabarba C, Dai LY, Oner FC. AO spine injury classification system: a revision proposal for the thoracic and lumbar spine. Eur Spine J. 2013;22(10):2184-201.

6. Magerl F, Aebi M, Gertzbein SD, Harms J, Nazarian S. A comprehensive classification of thoracic and lumbar injuries. Eur Spine J. 1994;3(4):184-201.

7. Frankel HL, Hancock DO, Hyslop G, Melzak J, Michaelis LS, Ungar GH, et al. The value of postural reduction in the initial management of closed injuries of the spine with paraplegia and tetraplegia. I. Paraplegia. 1969;7(3):179-92.
